# Single‐Cell ICP‐MS in Veterinary Research: Measuring Cisplatin Uptake in Healthy and Cancerous Animal Cell Lines

**DOI:** 10.1155/vmi/2049743

**Published:** 2025-12-11

**Authors:** Gábor Andócs, Csaba Kővágó, Julianna Flóra Szabó, László Könyves, Balázs Berlinger

**Affiliations:** ^1^ Department of Microbiology and Infectious Diseases, University of Veterinary Medicine, Budapest, Hungary, univet.hu; ^2^ Department of Pharmacology and Toxicology, University of Veterinary Medicine, Budapest, Hungary, univet.hu; ^3^ Department of Animal Hygiene, Herd Health, and Mobile Clinic, University of Veterinary Medicine, Budapest, Hungary, univet.hu

**Keywords:** 4T1, C26, carcinoma cell lines, cell fragmentation, MDCK

## Abstract

Platinum derivatives have been used in cancer treatment for several decades. However, the clinical effectiveness of these drugs is significantly hindered by their toxicity, resulting from accumulation in healthy cells and by the development of resistance in specific cancer cells. Previous research has successfully explored cisplatin’s mechanisms of cell transport, its antineoplastic effects, and its toxicity. Nevertheless, quantifying platinum uptake in individual cells posed a technological challenge until recent advancements. The single‐cell inductively coupled plasma mass spectrometry (SC ICP‐MS) method utilized in this study addresses this challenge. In our experiments, we used two murine carcinoma cell lines, C26 (colorectal carcinoma) and 4T1 (mammary carcinoma), along with a healthy epithelial cell line (MDCK) derived from a canine kidney. The cell cultures were exposed to various concentrations of cisplatin (10, 20, and 40 μM) for 24 h, followed by three washing steps and centrifugation. We monitored morphological changes in the cell cultures using an Olympus IX70 inverted phase‐contrast microscope, while cell counts were measured with a Merck Scepter 3.0 cell counter. The uptake of platinum and its intercellular distribution were assessed using a PerkinElmer NexION2000 ICP‐MS. Different cell lines absorbed platinum to varying degrees when exposed to the same cisplatin concentrations. Higher drug concentrations corresponded to increased amounts of platinum measured within all cell cultures. This relationship was directly proportional for several cell lines within specific concentration ranges. Notable cell death occurred in all cell line cultures when exposure exceeded a particular concentration, resulting in cell fragmentation. The SC ICP‐MS technique detected this as an increase in cell number. Our findings corroborate several previous studies and highlight the applicability of the SC ICP‐MS method in both human and animal cancer research.

## 1. Introduction

Neoplastic diseases are increasingly significant in both human and veterinary medicine, particularly among companion animals, where they are leading causes of death [1]. Epidemiological data indicate that 40%–50% of dogs over the age of 10 die from cancer [2]. Factors such as longer lifespans, selective breeding with associated genetic predispositions (e.g., histiocytosis in Bernese Mountain Dogs, osteosarcoma in Irish Wolfhounds, mastocytomas in Boxers) [3], and shared environmental carcinogens [4] contribute to this high incidence. Consequently, canine cancers display a diversity comparable to human cancers, often involving the same genetic mutations and showing similar responses to chemotherapeutic agents [5].

Cisplatin remains one of the most widely used platinum‐based chemotherapeutic agents, primarily for solid tumors such as sarcomas when radiotherapy is not viable, and as part of combination regimens for metastatic disease [6]. In veterinary practice, it has shown efficacy against several canine cancers, including osteosarcoma, urinary bladder transitional cell carcinoma, squamous cell carcinoma, melanoma, and mesothelioma, but it is contraindicated in cats due to fatal pulmonary edema. It is also used intralesionally in horses for squamous cell carcinoma and sarcoids [7]. However, current professional guidelines (e.g., 2016 AAHA) do not recommend cisplatin, favoring carboplatin because of its lower toxicity [8].

Platinum derivatives act mainly by binding to DNA and forming intra‐ and interstrand crosslinks that inhibit replication and induce apoptosis [9]. Their clinical use is limited by severe side effects, particularly nephrotoxicity, which results from accumulation in renal tubular epithelial cells. This process is influenced by oxidative stress, inflammatory responses, and active uptake via transporters such as OCT2 [10, 11].

The quantitative determination of platinum uptake has long been hampered by methodological limitations. Conventional approaches relied on enzymatic or acid digestion of cell populations followed by bulk analysis using atomic absorption spectroscopy (AAS) or inductively coupled plasma atomic emission spectrometry (ICP‐AES), providing only average concentrations without information on cell‐to‐cell variability. Recent advances introduced single‐cell inductively coupled plasma mass spectrometry (SC ICP‐MS), which allows direct measurement of metal content at the individual cell level. The technique, conceptually related to single‐particle ICP‐MS, relies on introducing intact cells into the plasma, where ion bursts generate signals proportional to the intracellular element concentration [12].

SC ICP‐MS has been applied to a wide range of unicellular systems, including human and other mammalian cell lines [13–16], bacteria [17, 18], yeast [19, 20], and algae [18, 21–23]. These studies have demonstrated its capability to quantify mineral elements, metal nanoparticles, and trace metals at the individual cell level. Importantly, the technique has already been used to investigate cisplatin uptake in human cancer models. Stephan et al. compared cisplatin‐sensitive and cisplatin‐resistant ovarian cancer cell lines (A2780 vs. A2780/CP70) and found higher accumulation in the sensitive cells [24]. Corte Rodríguez et al. further examined concentration‐dependent uptake [25], Lim et al. extended the approach to cisplatin, carboplatin, and oxaliplatin [26], and Galé et al. even quantified cisplatin at the subcellular level in isolated nuclei [27].

While most previous applications of SC ICP‐MS have focused on human models, it is equally important to evaluate platinum‐based drugs in veterinary‐relevant systems. For this reason, we selected three cell lines representing both oncological and toxicological contexts. The C26 mouse colorectal carcinoma line has long been used as a platinum‐sensitive cancer model [28]. The 4T1 murine mammary carcinoma line is a highly invasive model of triple‐negative breast cancer, reflecting many features of its human counterpart and known to respond to platinum drugs [29, 30]. Finally, the Madin‐Darby canine kidney (MDCK) line, derived from renal tubular epithelium, is a well‐established system for studying epithelial transport and nephrotoxicity, which is a major limitation of cisplatin therapy [31].

This study aims to apply SC ICP‐MS to quantify cisplatin uptake at the level of individual veterinary‐relevant cells. By combining cancer models (C26 and 4T1) with a nephrotoxicity‐sensitive epithelial model (MDCK), we sought to capture both therapeutic and toxicological aspects of platinum drug action. We hypothesized that (i) cisplatin uptake would increase in a dose‐dependent manner across all cell lines, and (ii) the extent of cell‐to‐cell variability would differ among models. As this work represents the first step of a broader research program, these models were also intended to help identify and address potential analytical challenges typical of newly applied SC techniques. This interdisciplinary approach demonstrates how a fast transient signal method can be adapted for veterinary oncology and toxicology, opening opportunities for future studies of platinum and other metal‐based drugs in animal health.

## 2. Materials and Methods

### 2.1. Utilized Cell Lines, Their Cultivation, and Preparation for Analysis

Three cell lines were used in this study: C26 mouse colorectal carcinoma, 4T1 murine mammary carcinoma, and MDCK epithelial cells. The choice of these models has been discussed in the Introduction, where their oncological and toxicological relevance is outlined. Here, we emphasize that these lines were available in our laboratory and are routinely used in other experimental contexts at our university, providing us with extensive prior experience and reliable background knowledge for their cultivation and handling. Authentication testing of the cell lines was not conducted because we lacked the necessary equipment and faced budget constraints.

Dulbecco’s modified Eagle’s medium (high glucose, Merck KGaA, Darmstadt, Germany) was used as the cell maintenance medium, supplemented with 10% calf serum (Capricorn Scientific GmbH, Ebsdorfergrund, Germany) and 1% antibiotic–antimycotic solution (Corning Inc., Corning, NY, USA). Before measurements, the cells were maintained in a 25‐cm^2^ standard cell culture flask (TPP Techno Plastic Products AG, Trasadingen, Switzerland). They were then transferred to standard six‐well plates (Sarstedt AG & Co. KG, Nümbrecht, Germany) and incubated for 24 h. The cells were then exposed to cisplatin for 24 h. The original cisplatin solution (Accord Healthcare Inc., Raleigh, NC, USA) contained 1 mg/mL of cisplatin. In the cell cultures, the concentrations of cisplatin ranged from 10 to 80 μM. After the incubation period, the adherent cultures were detached from the bottom of the plates using trypsin–EDTA (0.25% trypsin–2.21 mM EDTA) solution (Corning Inc., Corning, NY, USA) and suspended in fresh cell maintenance medium. The cell suspension was centrifuged at 1000 rpm for 2 min using a standard laboratory centrifuge (HERMLE Labortechnik GmbH, Wehingen, Germany). The settled cells were resuspended in 10 mL of phosphate‐buffered saline (PBS) solution, which was prepared using high‐purity water (18.2 MΩ cm) produced by Purite Select Fusion 160 BP water purification equipment (SUEZ Water Technologies & Solutions, Trevose, PA, USA): sodium chloride (NaCl), potassium chloride (KCl), potassium dihydrogen phosphate (KH_2_PO_4_), and sodium dihydrogen phosphate (Na_2_HPO_4_) (Merck KGaA, Darmstadt, Germany). The mixture was then centrifuged again. Before the SC ICP‐MS measurements, the cell concentration was adjusted to approximately 100,000 cells/mL according to the manufacturer’s recommendations by adding PBS. In an experiment designed to prevent cell fragmentation during SC ICP‐MS measurements, the cells were fixed using glutaraldehyde after being exposed to cisplatin. Glutaraldehyde was added [32] to the cell suspension at a concentration of 0.75% by weight during the second resuspension in PBS. After this, an additional step of centrifugation and resuspension was carried out.

### 2.2. Microscopic Analysis, Cell Counting, and SC ICP‐MS Measurement

The morphological characteristics of the cultured cells were examined using an Olympus IX70 (Olympus Corporation, Tokyo, Japan) inverted phase‐contrast microscope. Images of the cells were captured with a Euromex CMax Pro (Euromex Microscopen BV, Arnhem, The Netherlands) camera. In the initial experiments, cell counts were monitored with a Bürker chamber and later with a Merck Scepter 3.0 (Merck Millipore, Burlington, MA, USA) hand‐held cell counter.

To measure the uptake of cisplatin by the cells and analyze their distribution, we utilized a PerkinElmer NexION2000 (PerkinElmer Inc., Waltham, MA, USA) ICP‐MS equipped with a sample introduction system designed for SC measurements. This included a CytoNeb nebulizer (Elemental Scientific Inc., Omaha, NE, USA) and a quartz SC spray chamber. The most crucial instrumental conditions are shown in Table 1. Results were processed using Syngistix Single‐Cell Application Software (PerkinElmer Inc., Waltham, MA, USA). Before the measurements, the device was calibrated with PBS solutions containing dissolved Pt at 1, 2, and 4 ng/mL concentrations, prepared from a standard Pt solution at 1000 mg/L (VWR International, Radnor, PA, USA). To measure sample transport efficiency (TE) and to calibrate the instrument to measure the cell concentrations, we used a PBS solution prepared with a 100‐fold dilution of a colloidal solution of 50‐nm‐diameter gold nanoparticles, which had a concentration of 1.1 × 10^7^ particles/mL (PerkinElmer Inc., Shelton, CT, USA). This approach of using gold nanoparticles is a common practice for determining TE in SC ICP‐MS. However, we acknowledge that this procedure may not accurately reflect the behavior of larger particles such as whole cells (10–20 μm), for which the effective TE is usually lower. To verify the consistency of our approach, we also performed measurements with 30‐ and 100‐nm gold nanoparticles, which provided comparable results within the expected particle size range. Although direct TE determination for intact cells would be preferable, the AuNP‐based method provided a practical compromise in the present study. During sample introduction, the samples were placed in 50‐mL glass beakers and mixed using an ESP model magnetic stirrer (VELP Scientifica Srl, Usmate, Italy). Every SC ICP‐MS measurement was performed in triplicate. The software calculates the SC ICP‐MS method detection limit for Pt based on the counts for the dissolved Pt background and adds three times the standard deviation of the background signal.

**Table 1:** SC ICP‐MS conditions.

Parameter	Value
Sample uptake rate	10 μL/min
RF power	1600 W
Nebulizer gas (argon) flow^∗^	0.3–0.35 L/min
Makeup gas (argon) flow	0.7 L/min
Monitored platinum isotope	^195^Pt
Dwell time	50 µs
Sample analysis time	60 s
Sample transport efficiency^∗∗^	35%–50%
Detection limit^∗∗^	0.008–0.018 fg Pt/particle or cell

^∗^It was adjusted each measurement day.

^∗∗^It varied by measurement day.

The bulk Pt concentrations in exposed and washed cell cultures were measured in an experiment. The final suspensions in PBS, which contained C26 cells exposed to cisplatin concentrations of 10, 20, 40, and 80 μM, were directly introduced into the ICP‐MS instrument. The instrument operated under “normal” conditions and was calibrated using PBS solutions with dissolved Pt at concentrations of 1, 5, and 25 ng/mL, with bismuth (Bi) as the internal standard at a concentration of 10 ng/mL.

## 3. Results and Discussion

### 3.1. Evaluation of the Applicability of the SC ICP‐MS Method for the Measurement of Cisplatin Uptake by Animal Cell Lines

Upon reviewing the measurement results from the initial experiments with cisplatin, it became evident that SC ICP‐MS detected increasing cell concentrations correlated with higher cisplatin exposure. This phenomenon can be partly explained by higher exposure levels, which result in more cells reaching the detection limit for Pt. However, the cell counts calculated by the software were often unrealistically high, particularly for cultures treated with 40 μM cisplatin. Bürker chamber cell counting performed on control samples confirmed that the concentration values determined by SC ICP‐MS were inaccurate at these higher exposure levels.

Understanding the device’s operating principle, we concluded that cell fragmentation was the most likely explanation for these observations. To prevent the potential disintegration of the cells during the SC ICP‐MS sample introduction, we fixed the cell cultures with glutaraldehyde, following literature recommendations [32]. In this follow‐up experiment, we found no significant difference in the measured cell numbers between samples exposed to the same concentration of cisplatin and containing approximately equal numbers of cells. This suggests that fragmentation likely occurred primarily during exposure to the Pt compounds. Microscopic examinations later corroborated this suggestion.

Thus, the apparent increase in the number of cells measured by SC ICP‐MS can be attributed to the cytotoxic effects of Pt. The observed cell count represents the sum of intact cells and cell fragments. In our final experiments, we utilized the Scepter 3.0 hand‐held cell counter to differentiate between intact cells and cell fragments. This device counts particles in different size ranges and displays the number of particles in each size fraction using histograms.

The uptake of cisplatin by C26 cells was assessed at three different concentration levels across completely independent experiments. We did not conduct multiple parallel treatments from the same culture. Each experiment began with a fresh cell culture. In one instance, when the cells were fixed, the sample preparation varied as well. The results from the independent experiments are summarized in Table 2. For the most frequent mass and median mass values, no error term is reported. These parameters are calculated automatically by the instrument software using the selected bin size (0.1 fg in this study). As a result, parallel measurements typically produced identical values, with occasional small differences when a result fell into an adjacent bin. Such bin‐size effects do not reflect analytical variability but are inherent to the algorithm used for calculating these parameters.

**Table 2:** Results from the SC ICP‐MS analysis (*n* = 3), uptake of cisplatin by C26 cells on independent experimental days.

Cisplatin exposure level (µM)	Experimental day	Most frequent mass (fg Pt/cell)	Mean mass (fg Pt/cell)	Median mass (fg Pt/cell)	Detection limit (fg Pt/cell)	Cell concentration (cell/mL)
10	Day 1	0.11	0.37 ± 0.06	0.21	0.010	6.2E + 04 ± 8E + 03
	Day 2^∗^	0.01	0.52 ± 0.03	0.21	0.008	1.5E + 05 ± 2E + 04
	Day 3	0.12	0.60 ± 0.3	0.32	0.015	3.5E + 04 ± 2E + 03

20	Day 1	0.21	0.58 ± 0.03	0.31	0.010	2.3E + 05 ± 2E + 04
	Day 2^∗^	0.21	1.09 ± 0.03	0.41	0.008	4.2E + 05 ± 2E + 04
	Day 3	0.32	0.69 ± 0.05	0.42	0.015	1.5E + 05 ± 9E + 03

40	Day 4	0.32	0.92 ± 0.04	0.42	0.018	5.3E + 05 ± 2E + 04
	Day 2^∗^	0.21	1.79 ± 0.04	0.41	0.008	5.1E + 05 ± 4E + 04
	Day 3	0.42	1.36 ± 0.13	0.52	0.015	4.1E + 05 ± 5E + 04

^∗^The cells were fixed with glutaraldehyde.

The data obtained from three independent experiments were evaluated statistically [33]; however, the limited number of replicates did not allow for robust statistical analysis. Therefore, we present the results as simple comparative evaluations, highlighting reproducible trends rather than formal statistical significance testing.

Our findings indicate a clear relationship between cisplatin exposure and Pt uptake in C26 cells. Higher concentrations of cisplatin consistently resulted in increased levels of Pt accumulation within the cells on each experimental day. Specifically, when we examined the effects of double Pt exposure, we observed that the amount of Pt in the cells—as well as in cell fragments in case of 20 μM or higher cisplatin exposures—doubled, indicating a strong dose–response relationship. However, there was a considerable variation in the number of cells detected, which was influenced by cell fragmentation. The results from parallel experiments using SC ICP‐MS conducted on the same experimental day showed good consistency; the average amount of Pt uptake did not exhibit large fluctuations, and the relative standard deviation (RSD) typically remained below 10%. However, we did observe more pronounced differences in results across different measurement days, with RSD values ranging from 20% to 35%. This variability is consistent with findings reported in existing literature [25, 26] and reflects a common phenomenon in *in vitro* experimental settings. We also noted minor differences in median masses across different experimental days.

In an experiment, we conducted bulk ICP‐MS and SC ICP‐MS analyses of the same cell suspensions containing C26 cells. We found no considerable difference in the Pt background concentrations measured by SC ICP‐MS compared to the average Pt concentration in the whole‐cell suspensions, including the cells (Table 3). The ability to determine the Pt content of individual cells—often cell fragments—in suspensions with relatively high background Pt concentrations demonstrates that SC ICP‐MS offers superior performance and accuracy over the conventional ICP‐MS method. With the bulk method, the background contribution also affects the average intracellular Pt concentrations. Although increasing the number of washing steps can further reduce background, it may introduce additional errors during sample preparation and increase processing time.

**Table 3:** Comparison of the SC ICP‐MS and “bulk” ICP‐MS methods.

Cisplatin treatment level (µM)	Pt background concentrations determined by SC ICP‐MS (ng/mL)	Average Pt concentration in the washed cell suspension^∗^ (ng/mL)	Mean Pt mass in the cells/cell fragments^∗∗^ (fg)
10	0.57 ± 0.03	0.53 ± 0.02	0.28 ± 0.04
20	1.04 ± 0.03	1.74 ± 0.03	0.51 ± 0.04
40	1.86 ± 0.07	2.85 ± 0.05	0.92 ± 0.04
80	4.55 ± 0.30	8.00 ± 0.14	2.14 ± 0.43

^∗^Measured by ICP‐MS.

^∗∗^Measured by SC ICP‐MS.

### 3.2. The Uptake of Cisplatin by the Different Cell Lines

**Figure 1:**
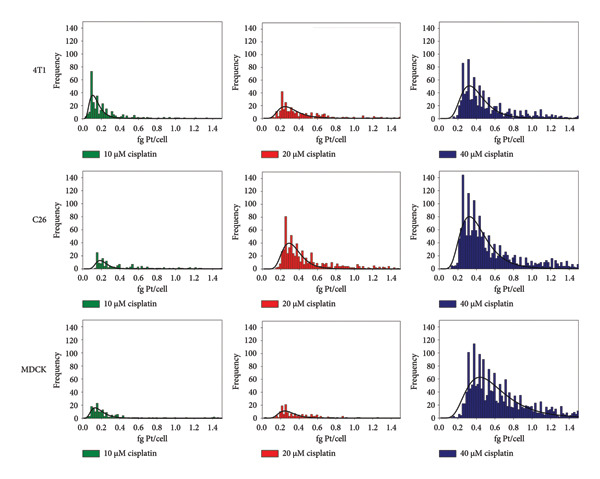
Frequency distributions of intracellular platinum masses measured by SC ICP‐MS in 4T1, C26, and MDCK cells exposed to different concentrations of cisplatin (10–40 μM).

In Figure 1, the Pt uptake in cells treated with cisplatin is illustrated using histograms, with 3‐parameter lognormal curves fitted to the data. Table 4 provides essential information obtained from SC ICP‐MS measurements regarding Pt uptake by the cells, including the most frequently recorded, average, and median masses. For the most frequent and median values, no error terms are reported, as noted above.

**Table 4:** Results from the SC ICP‐MS analysis (*n* = 3), uptake of cisplatin by different cell lines.

Cell line	Cisplatin exposure level (µM)	Most frequent mass (fg Pt/cell)	Mean mass (fg Pt/cell)	Median mass (fg Pt/cell)	Cell concentration (cell/mL)
4T1	10	0.02	0.32 ± 0.07	0.12	5.8E + 04 ± 6E + 03
	20	0.22	0.63 ± 0.06	0.32	6.5E + 04 ± 1E + 03
	40	0.32	0.73 ± 0.09	0.42	1.8E + 05 ± 3E + 04

C26	10	0.12	0.60 ± 0.03	0.32	3.5E + 04 ± 2E + 03
	20	0.32	0.69 ± 0.05	0.42	1.5E + 05 ± 9E + 03
	40	0.42	1.36 ± 0.13	0.52	4.1E + 05 ± 5E + 04

MDCK	10	0.02	0.43 ± 0.03	0.22	4.1E + 04 ± 8E + 03
	20	0.22	0.83 ± 0.05	0.42	3.4E + 04 ± 9E + 02
	40	0.52	1.85 ± 0.14	0.72	4.4E + 05 ± 3E + 04

The results from three parallel SC ICP‐MS measurements on the same cell line exposed to identical concentrations of cisplatin showed minimal variance. The RSDs for the average of Pt mean masses taken up by the cells and cell concentrations were generally less than 10%. As expected, the amount of Pt within the cells increased with higher concentrations of cisplatin, with different cell lines exhibiting varying degrees of Pt uptake at various exposure levels (see Figure 1). The differences in average Pt mass among the various cell types were generally low. Notably, the 4T1 cells displayed a lower average Pt content at all exposure levels compared to the other two cell types.

In most cases, there was a discrepancy between the number of cells detected by the SC ICP‐MS method and those counted by the cell counter. One reason for the lower cell concentrations observed with the SC ICP‐MS method is that the software did not account for cells that absorbed Pt below its detection limit. Notably, in the 4T1 and MDCK cell lines, a sudden increase in cell concentration was observed at 40 μM exposure to cisplatin, while in the C26 cell line, this increase occurred already at 20 μM exposure. This phenomenon may be attributed to the cytotoxic effects of cisplatin, which can lead to cellular fragmentation. The measurement system detected the cell fragments as individual particles. The significant rise in the number of particles detected in the sub‐5 μm range by the cell counter, in conjunction with increasing cisplatin exposure, supports the fragmentation hypothesis. However, assuming that the cell fragments contained less Pt than the still intact cells is reasonable. This assumption is particularly evident in the 4T1 and C26 cell lines, where the sudden increase in apparent cell concentration did not correspond with a proportional increase in average Pt uptake.

For the 4T1 cell line, increasing the cisplatin exposure from 10 to 20 μM resulted in a directly proportional increase in cisplatin uptake. The nearly identical cell concentrations measured at 10 and 20 μM, which were not significantly different from those recorded by the cell counter, suggest that cell fragmentation had not yet occurred at these levels. In contrast, the linear increase in average intracellular Pt concentration observed in the C26 and MDCK cells at higher cisplatin exposures—considering cell fragmentation—indicates that these cells likely absorbed the Pt complex almost uncontrollably at such concentrations. This behavior might be due to disrupted transport mechanisms in damaged cells, preventing them from effectively removing the intracellular Pt through active transport.

**Figure 2:**
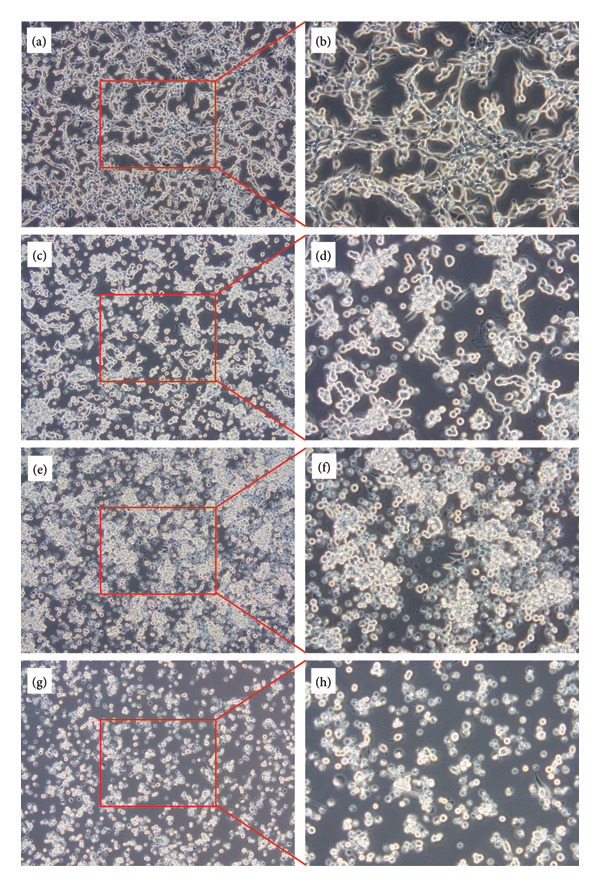
Morphological image of C26 cells after 24‐h exposure to cisplatin at 100 × and 200 × magnification. (a, b: untreated control, c, d: 10 μM cisplatin, e, f: 20 μM cisplatin, and g, h: 40 μM cisplatin).

Based on the data measured by SC ICP‐MS and the histograms, all cell lines showed that increased exposure led to either a higher number of cells taking up measurable amounts of Pt, greater amounts of Pt being absorbed by individual cells, or both. Our findings confirm those of previous studies [25–27]. The average amount of Pt taken up by the cells was also consistent in magnitude. However, it is important to note that different cell lines were used in prior experiments. SC ICP‐MS has not yet been employed to investigate the uptake of Pt complexes in the cell lines we utilized. The histograms do not clearly indicate whether the cells’ Pt uptake follows a normal or lognormal distribution; however, lognormal curves fit the distributions effectively. Notably, in all instances, some cells or cell fragments—despite being in negligible amounts—showed a significantly higher Pt uptake than the average. The amount of Pt accumulated within the cells was indicative of cell death, demonstrating that the cytotoxic effects of Pt compounds depend significantly on how efficiently they enter the cells [9]. Nevertheless, previous studies did not report notable cell fragmentation in response to cisplatin treatment. This lack of fragmentation might be attributed to using lower concentrations of cisplatin [25] and/or shorter exposure times [26]. In contrast, our microscopic examinations also clearly exhibited cell fragmentation due to cisplatin exposure. The microscopic images reveal that untreated C26 cells adhere to the bottom of the plate, are spindle‐shaped, and do not form a uniform monolayer. After cisplatin treatment, the cells round up and detach from the substrate in a concentration‐dependent manner, appearing as spheres floating in the medium. Significant cell death is observed in cultures exposed to 40 μM cisplatin, resulting in numerous bright, apoptotic cells and debris particles smaller than the cells (Figure 2).

A limitation of the detailed evaluation of results is that SC ICP‐MS is primarily suitable for detecting differences in Pt uptake across various exposure levels and cell lines. It does not provide insights into the underlying causes of these differences. Various transport mechanisms [34–36] and structural changes in the cell membrane [37–40] likely play significant roles in the uptake of Pt complexes, cell damage, and potential resistance. However, investigating these factors requires the use of techniques beyond SC ICP‐MS.

## 4. Conclusions

When applying SC ICP‐MS in examining cisplatin uptake by three cell lines, we made the following observations: (1) The SC ICP‐MS technique is effective for studying the uptake and distribution of cisplatin by cells and offers several advantages compared to traditional bulk methods. However, other techniques, such as microscopy and cell counting, are also necessary to evaluate the results. (2) Different cell lines generally absorbed the complexes to varying degrees, as demonstrated by the differing amounts of Pt measured within the cells. (3) Cells exposed to higher concentrations of cisplatin exhibited greater uptake of the cytostatic. For several cell lines, this relationship was directly proportional within specific concentration ranges. (4) We noted a significant level of cell death above a certain concentration in all cell lines. This phenomenon resulted in a high degree of cellular fragmentation, which led to the detection of an increased number of cells/cell fragments using the SC ICP‐MS method.

These findings were generally consistent with previously published results. Although the methods we employed do not allow for the explanation of the observed relationships regarding cellular processes, the quantifiable results from our research, alongside similar studies investigating the uptake of other complexes, may contribute to the ongoing development of both human and animal cancer therapies utilizing metal complexes in clinical practice.

## Conflicts of Interest

The authors declare no conflicts of interest.

## Funding

This study was funded by the Strategic Research Fund of the University of Veterinary Medicine Budapest (Grant Nos. SRF‐001, SRF‐002). The cell counter used in this study was purchased with the support of the Strategic Research Fund of the University of Veterinary Medicine Budapest (Grant No. SRF‐002). The János Bolyai Research Scholarship from the Hungarian Academy of Sciences supported this study.

## Data Availability

The data that support the findings of this study are available from the corresponding author upon reasonable request.
